# Cerebral and Nerve Surgery

**Published:** 1904-10-08

**Authors:** 


### CEREBRAL AND NERVE SURGERY.

The Surgery of the Gasserian Ganglion.[1]—After pointing out that all operations on the branches of the fifth nerve or their ganglia distal to the Gasserian ganglion performed for the relief of neuralgia, result in more or less rapid recurrence of the symptoms, Armour proceeds to describe the various methods of reaching and excising the intracranial portions of the trigeminal nerve. Rose's method in which the zygoma and coronoid process are resected and the ganglion reached through the pterygoid fossa; the Hartley Krause method in which a large opening is made in the temporal fossa and the ganglion reached by turning up the temporal lobe of the brain. Doyen's and Cushings' methods which are compromises between the foregoing, involving resection of the zygoma and coronoid and an approach to the ganglion through the lowest part of the temporal fossa; Ramonede's method attacks the nerve between the pons and ganglion by the occipital route, the bone opening being made just within the arch of the sigmoid sinus. It is asserted that the mortality of the operation which stood at 30 per cent. on its introduction has been reduced to $2\frac{1}{2}$ per cent. in the series of 120 cases performed by Sir Victor Horsley. Also it is maintained that in no single case where the ganglion has been removed *in toto* has there been any return of the pain.

Surgery of the Fifth Nerve.—J. Hutchinson, jun.[2] describes a case of long continued and intractable neuralgia confined to the second division of the fifth nerve in which he resected the intra-cranial portion of this division between the Gasserian ganglion and the foramen rotundum. The nerve was reached by the same route as that employed in the Hartley Krause operation on the Gasserian ganglion.

Laminectomy for Injuries to the Spinal Cord.—Mixter and Chase[3] describe two cases of fracture of the cervical spine which had all the symptoms of total transverse lesion of the cord, and in which, nevertheless, the functions of the cord were more or less completely restored after laminectomy. In the first case, a man of 40, the laminæ of the fourth, fifth, and sixth vertebræ were fractured and depressed. Complete paralysis and loss of tactile sensation and reflexes were present below the distribution of the sixth cervical nerve. Operation within 24 hours. Sensation returned in six days, the reflexes in 12, motion partly in three months, more fully in six months; died $11\frac{1}{2}$ months after operation from suppurative nephrolithiasis. Sections of the cord showed degeneration tracts of sensory nerves upwards, and motor nerves downwards, but also they showed that many neurons had been preserved intact in the region of the lesion. In the second case a man of 27 had a fracture and depression of the sixth and seventh cervical laminæ; paralysis, anæsthesia, and loss of reflexes were complete below the lesion; operation was performed within two days. Sensation and reflexes had returned within 12 days, together with feeble movements. Motion had been restored partially to all limbs within four months, and improvement was noticed during the whole of the five years he was under observation. These cases presented all the symptoms of total transverse lesion of the cord, including the loss of reflexes, and yet they both made great improvement after operation. It is urged that no delay beyond a few hours for the subsidence of shock be allowed before operating, as in one recorded case where death occurred on the fifth day marked degeneration was found.

**Tumours of the Brain.**—Frazier[4] in discussing the surgery of brain tumours emphasises the following points: the importance of avoiding shock by shortening the operation, preventing hæmorrhage, and not manipulating the brain more than necessary, the value of the electric engine in opening the skull and exposing large areas of brain, the great value of observations on the blood-pressure before and during the operation as indicating the patient's condition, and the necessity of doing the operation in two stages, the difficulties caused by cerebral bulging, the "initial bulging" being caused by the pressure of the tumour and "consecutive bulging" by cerebral œdema set up by operative manipulations, and lastly the importance and necessity of palliative operations.

**Fractures of the Skull.**—Mr. L. B. Rawling[5] lays great stress on the structure of the skull, and its lines of greatest strength bounding areas of weakness in determining the course of fractures. He considers that most basic fractures result from direct irradiation from forces applied to the vault. A detailed examination of the influence of the various foramina and sinuses in determining the direction and extent of basic fractures, leads to the conclusion that if the point of application and direction of the fracturing force be known, then the position and direction of the resulting fracture can be accurately predicted. Hæmorrhages resulting from cranial fractures may be external or internal. Of external hæmorrhages much the most important is that from the external auditory meatus. When this is profuse it generally is the result of a laceration of the internal meningeal artery. Severe hæmorrhage from the nose, or into the naso-pharynx, is due to a fracture of the body of the sphenoid, lacerating the cavernous sinus, or even the internal carotid artery. The pathology and symptoms of the various forms of intra-cranial hæmorrhage and of the lesions of the cranial nerves resulting from fracture are entered into in detail. The fatal results of cerebral compression are related to pressure on the bulbar nuclei, and it is proposed that in desperate cases the posterior fossa should be trephined in order to relieve this condition.

**Acute Osteomyelitis of the Spine and Acute Suppurative Perimeningitis.**—Dr. Ramsay Hunt[6] describes two cases of the former affection, and collects and discusses 62 cases from the literature. It is a disease most common in young adults; it generally follows a blow, and is caused by an invasion of the staphylococcus pyogenes in over 80 per cent. It may attack the bodies or laminæ of the vertebræ. In the former case the resultant abscess will point in the pharynx, mediastium, or psoas muscle, and in the latter over the spine posteriorly. In about one-third of the cases an extra-dural abscess of the spine, with resulting pressure symptoms, was produced. With regard to the so called suppurative perimeningitis, 13 out of the 24 published cases in which sufficient detail is given are analysed, and it is shown that in all these the lesion was probably primary in the bone, and constituted an osteomyelitis with secondary involvement of the spinal meninges.

[1] Practitioner, p. 898. [2] Lancet, April 16. [3] Annals of Surgery, April. [4] Amer. Jour. Med. Sci., February. [5] Lancet, April 9-23. [6] Med. Record, April 23.

